# Is there less to social anxiety than meets the eye? Behavioral and neural responses to three socio-emotional tasks

**DOI:** 10.1186/2045-5380-3-5

**Published:** 2013-03-01

**Authors:** Michal Ziv, Philippe R Goldin, Hooria Jazaieri, Kevin S Hahn, James J Gross

**Affiliations:** 1 Department of Psychology, Stanford University, Jordan Hall, Bldg. 420, Stanford, CA, USA

**Keywords:** Social anxiety, Amygdala, Insula, Emotion reactivity, fMRI, Socio-emotional tasks

## Abstract

**Background:**

Social anxiety disorder (SAD) is widely thought to be characterized by heightened behavioral and limbic reactivity to socio-emotional stimuli. However, although behavioral findings are clear, neural findings are surprisingly mixed.

**Methods:**

Using functional magnetic resonance imaging (fMRI), we examined behavioral and brain responses in *a priori* emotion generative regions of interest (amygdala and insula) in 67 patients with generalized SAD and in 28 healthy controls (HC) during three distinct socio-emotional tasks. We administered these socio-emotional tasks during one fMRI scanning session: 1) looming harsh faces (Faces); 2) videotaped actors delivering social criticism (Criticism); and 3) written negative self-beliefs (Beliefs).

**Results:**

In each task, SAD patients reported heightened negative emotion, compared to HC. There were, however, no SAD versus HC differential brain responses in the amygdala and insula. Between-group whole-brain analyses confirmed no group differences in the responses of the amygdala and insula, and indicated different brain networks activated during each of the tasks. In SAD participants, social anxiety symptom severity was associated with increased BOLD signal in the left insula during the Faces task.

**Conclusions:**

The similar responses in amygdala and insula in SAD and HC participants suggest that heightened negative emotion responses reported by patients with SAD may be related to dysfunction in higher cognitive processes (e.g., distorted appraisal, attention biases, or ineffective cognitive reappraisal). In addition, the findings of this study emphasize the differential effects of socio-emotional experimental tasks.

## Background

Social anxiety disorder (SAD) is defined by a marked and persistent fear of social or performance situations in which the person is exposed to unfamiliar people, or possible scrutiny by others [1]. When interacting with others, people with SAD experience intense anxiety accompanied by physical symptoms such as blushing, heart racing, sweating, trembling, and nausea, as well as stuttering and trouble concentrating [2]. SAD is one of the most common anxiety disorders, with a lifetime prevalence of up to 13% [3]. When untreated, SAD can lead to elevated social isolation, depression, anxiety, and alcohol and/or substance abuse [4].

Models of SAD have highlighted the role of emotional hyper-reactivity in feared social situations. Exaggerated emotional responses are thought to arise from maladaptive appraisals of the social situation that transform innocuous social cues into interpersonal threats [5]. This hyper-reactivity often leads to attempts to escape from or avoid the anxiety-provoking object or situation [2].

To study the behavioral and brain aspects of this emotional hyper-reactivity, most studies have employed negatively valenced facial expressions as socio-emotional probes. Behaviorally, these studies show hyper-arousal and increased emotional distress in patients with SAD when exposed to negative face stimuli [2]. Neurally, however, the findings are less clear.

The amygdala, which plays a critical role in the recognition of fear in facial expressions [6], was a natural target in studies of the neural basis of emotional reactivity in SAD. It was suggested that the heightened emotional responses in SAD patients were associated with increased amygdala activity [7]. Amaral [8] proposed a model based on lesion studies in monkeys, which suggested that the amygdala performs a protective function, namely, allowing the organism to detect and avoid danger. This model provided a rationale for theories implicating the amygdala in the neuropathology of SAD.

Many studies have indeed demonstrated heightened amygdala responses in patients with SAD when viewing angry, fearful and even neutral facial expressions [7,9-12]. However, other studies, some using stimuli other than faces, failed to detect differential amygdala activation between patients with SAD and HC [13-17]. For example, Quadflieg and colleagues (2008) have found that both SAD patients and HC recruit the amygdala when listening to angry compared to neutral prosody [17]. Similarly, Goldin and colleagues (2009) found increased amygdala responses to harsh facial expressions with longer stimulus presentation times (6 seconds) in both SAD patients and healthy controls [16]. Other studies have even found *decreased* amygdala responses in SAD patients compared to HC. For example, Kilts and colleagues (2006) have found decreased amygdala responses during both script-driven imagery of anxiety-provoking social situations and a mental arithmetic task in SAD [14].

A second limbic region that is thought to play a prominent role in SAD neuropathology is the insula. The insula is interconnected with the amygdala, thalamus, and orbitofrontal cortex [18], regulates the autonomic nervous system [19], and is involved in the recognition and experience of aversive states [20] and threat processing [21]. Thus, hyper-reactivity of the insula in SAD patients might be responsible for the generation of fear responses to social aversive stimuli.

A recent meta-analysis identified four functionally distinct sub-regions in the human insula implicated in social-emotional, sensorimotor, olfacto-gustatory, and cognitive brain networks [22]. The ventral part of the insula was associated with the social-emotional domain. This ventral region was active in 195 experiments of emotion generation (paradigms of “induction, imagination or recall of own happiness, fear, anxiety, anger, sadness, or disgust”) and 120 empathy related experiments (paradigms of judging emotions in faces or attending to pain in others). This differentiation matches previous cytoarchitectonic studies in non-human primates that found differences between the anterior-basal agranular, allocortical, and posterior granular parts of the insula [23]. The anterior-ventral region is involved in the generation and mediation of feeling as a response to environmental stimuli and affective states [24], and in the recognition of emotions in faces [25]. In SAD, increased activity in this insula region was associated with visualization of angry and disgusted faces [13,26], viewing of images with negative emotional content [27], and anticipation of a public speaking task [28]. However, several studies did not find such activity differences [17,29] and others found *decreased* activity in the insula during a public speaking task [30] and during an implicit learning test [31].

Thus, despite the clear focus of neuroimaging studies on the amygdala and insula as key regions in SAD pathology, findings are mixed. One explanation for these mixed findings might be related to factors such as different stimuli and experimental designs used to elicit emotional responses (mainly emotional faces until a few years ago, and images, written words, and auditory stimuli more recently), small patients groups, differences in exclusion criteria (e.g., different psychiatric co-morbidity), and other experimental parameters.

However, another explanation could be a lack of concordance between the behavioral and physiological components of anxiety. This idea was raised by Mauss and colleagues in a study of non-clinical social anxiety [32]. In this study, anxiety experience, subjectively perceived physiological activation, and objectively measured physiological activation were assessed in high and low trait social anxiety participants using an impromptu speech paradigm. The study found no differences in physiological responding between high and low trait social anxiety, despite clear differences between the two groups in their subjective experience of anxiety and their perceived physiological activation. Although this study did not measure brain responses, and did not employ a clinical sample, it did raise an important question about the widely assumed link between behavioral and physiological responses in the context of social anxiety.

In light of the mixed literature on the neural correlates of emotion reactivity in SAD, the goal of the present study was to examine both behavioral responses and BOLD signal changes in SAD compared to HC when reacting to distinct types of socio-emotional stimuli. To test whether behavior and brain responses vary by context, we used three different socio-emotional tasks: 1) viewing looming harsh faces (Faces), 2) watching dynamic videos of actors delivering social criticism (Criticism), and 3) reading negative self-beliefs (Beliefs). These tasks mirror the specific types of interpersonal evaluations and negative thoughts that are especially salient in patients with SAD. As suggested by the literature, we focused on the amygdala and the insula regions, but also performed whole-brain analyses to examine the involvement of other brain networks in SAD.

We hypothesized that, compared to HC, patients with SAD would have (1) greater self-reported negative emotion reactivity for each of the three tasks; and (2) greater BOLD responses in the amygdala and insula. In secondary analyses, we examined the differential effect of each socio-emotional task on negative emotion reactivity and neural responses in SAD and HC, and whether – among SAD participants – negative emotion reactivity and neural responses were associated with social anxiety symptom severity.

## Methods

### Participants

Participants included 67 (32 females) adults who met *DSM-IV-TR*[1] criteria for primary generalized SAD and 28 (13 females) healthy controls (HC) with no lifetime history of psychiatric disorders (Table 1). Patients were recruited through clinician referrals and advertisements on community and online bulletin boards. Two PhD-level clinical psychologists assessed each potential participant using the Anxiety Disorders Interview Schedule for *DSM-IV-TR Lifetime version (ADIS-IV-L*) [33]*.* We invited into the study only patients who met clinical diagnostic criteria for a principal diagnosis of current generalized SAD (defined as greater than moderate anxiety/fear for 5 or more distinct social situations) or healthy controls (with no current or past history of DSM-IV disorders).

**Table 1:** Demographic and clinical variables

	**SAD n = 67**	**HC n = 28**	***t*-value**	**Partial eta**^**2**^
Females (n)	32	13		
Age (Mean years ± SD)	33.0 ± 8.8	32.6 ± 9.5	0.2	
Education (Mean years ± SD)	16.7 ± 2.4	17.5 ± 1.5	1.6	
EDI (Mean ± SD)	9.9 ± 0.3	9.8 ± 0.4	0.30	
Ethnicity				
- Caucasian	38	17		
- Asian	17	8		
- Latino	8	2		
- Native American	1	1		
- Native Hawaiian	1	0		
- Filipino	1	0		
LSAS-SR (Mean ± SD)	84.1 ± 17.5	15.3 ± 9.1	19.3 *	.85

Note: EDI = Edinburgh Handedness Inventory, LSAS-SR = Liebowitz Social Anxiety Scale–Self-Report.

* *P* < .0001.

Both SAD and HC had a mean age of 33 years (range: SAD 21–53 years; HC 21–52 years, see Table 1) and were right-handed as assessed by the Edinburgh Handedness Inventory [34]. Potential patients were excluded if they reported current pharmacotherapy or psychotherapy, history of neurological disorders, and current psychiatric disorders (other than SAD, generalized anxiety disorder, agoraphobia without a history of panic attacks, dysthymia, or specific phobia). HC were not permitted to meet criteria for any current or past psychiatric disorders.

Among patients, *current* Axis-I co-morbidity included 3 with panic attacks, 13 with generalized anxiety disorder, 3 with dysthymia and 5 with specific phobia. *Past* Axis-I co-morbidity included 13 with major depression, 4 with substance abuse, 1 with panic attacks, 1 with dysthymia, and 1 with eating disorder. Thirty-three patients reported past (i.e., ended more than 1 year ago) non-cognitive-behavioral psychotherapy, and 20 reported past pharmacotherapy. All participants provided informed consent in accordance with Stanford University Human Subjects Committee rules.

### Clinical assessment

To asses social anxiety symptom severity, participants completed the Liebowitz Social Anxiety Scale-Self-Report (LSAS-SR) [35]. This questionnaire assesses both fear and behavioral avoidance of social situations, and is widely used in the research of SAD [36].

### Experimental tasks

Prior to scanning, patients were trained on the three socio-emotional tasks programmed in Eprime (Psychology Software Tools, Inc). The stimuli used in the pre-fMRI training were not the same stimuli used in the MR scanner. All three tasks were composed of “React”, “Asterisks” and “Reframe” conditions. In the present study, we report on data from the React condition, contrasting it with the Asterisks condition. The results for the Reframe condition will be published elsewhere.

During the “React” condition, participants were instructed to react normally without any attempt to control, modify, or regulate any reactions. After each React condition, participants provided a negative emotion rating using a button response pad positioned in the participant’s right hand inside the magnet by responding to “How negative do you feel?” (1 = not at all to 5 = very much).

The Asterisks condition served as a low-level comparison condition, during which one to five white asterisks were displayed over a black background. Participants were asked to count how many asterisks were on the screen during the Faces and Criticism tasks, and were not given any counting instructions during the Beliefs task. There were 13 trials for this condition in the Faces task, 18 trials in the Criticism task, and 7 trials in the Beliefs task.

#### Harsh looming faces (Faces task)

**Figure 1:**
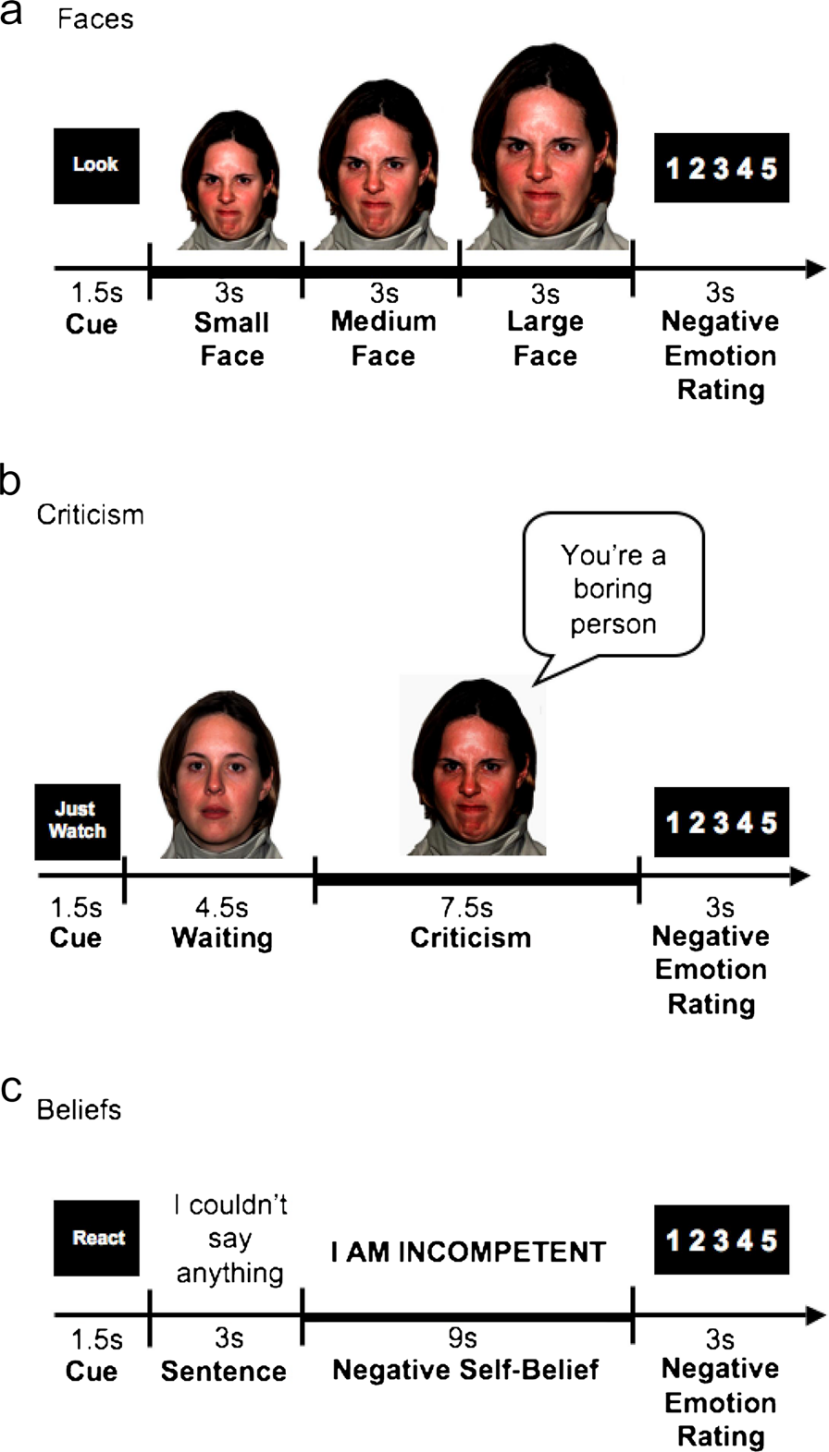
**The three socio-emotional tasks. **(**a**) Harsh looming faces (Faces); (**b**) social criticism (Criticism); (**c**) negative self-beliefs (Beliefs). For each task, a “React” trial consisted of: 1) a 1.5 seconds “Cue”; 2) a socio-emotional stimulus (a harsh looming face/ a video-clip of an actor delivering social criticism/ an autobiographical sentence + NSB); and 3) a negative emotion rating scale. Participants were instructed to react normally to the stimuli without any attempt to control, modify or regulate their reactions.

The task consisted of 24 trials during which the participant viewed color photos of Ekman’s facial action coded [37] faces displaying anger and contempt. The length of the entire task was 516 TRs, which is 12 minutes and 54 sec (774 sec). Each trial consisted of a cue to look (1.5 sec), a single harsh facial stimulus (9 sec) presented in color and appearing to move closer to the participant every 3 sec to simulate looming, and a negative emotion rating after the face stimulus terminated (3 sec) (Figure 1a). Participants were trained prior to the baseline scan to react to the faces by engaging in the picture (“Just let yourself feel”) and thinking: "This person is upset with me; angry with me”.

#### Dynamic social criticism (Criticism task)

The task consisted of videotaped actors delivering social criticism and social praise and harsh or happy evaluation-congruent facial expressions (Figure 1b). The stimuli were delivered by five male and five female actors (seven Anglo-Americans and three Asian-Americans) with an age range of 23–50. Each condition consisted of 16 trials delivered across two runs of 342 TRs, 8 min 35 sec each (513 sec). Each 13.5 sec trial consisted of a cue to “Just Watch” (1.5 sec), followed by a video clip of an actor delivering social evaluation (12 sec). Each 12 sec video clip had a 4.5 sec waiting period during which the actor silently maintained a neutral facial expression followed by a 7.5 sec evaluation period in which the actor delivered a social criticism or praise statement while displaying a harsh or positive facial expression. For the current study, only social criticism trials were included in the analysis (see examples in Table 2). After each video clip, participants were cued to rate their current negative emotion (3 sec). Participants were trained prior to scanning to react to the social criticism by reflecting on how the statement represents something true about themselves.

**Table 2:** Examples of social criticism and negative self-beliefs

**Social criticism statements**	**Negative self beliefs**
You don't seem very smart	I ALWAYS MESS UP
You've lost my interest	OTHERS SEE MY FAULTS
I feel uncomfortable being with you	I FELT HUMILIATED
You would not make a good leader	OTHERS THINK I'M A FOOL
You don't seem courageous	I AM INCOMPETENT
You're a boring person	OTHERS DO NOT LIKE ME
Do you always look so run down?	I AM WEIRD
You haven't accomplished much	OTHERS THINK I AM NOT NORMAL
Everyone thinks you are so weird	I AM STUPID
You never know what to say	OTHERS KNOW SOMETHING IS WRONG WITH ME

#### Negative self-beliefs (Beliefs task)

This task consisted of five situations. The first was an experimenter-composed neutral situation about cleaning a car that was used to obtain baseline emotion ratings for reading neutral statements. Then, four participant-generated autobiographical social anxiety situations characterized by social anxiety, humiliation, and embarrassment were presented. Three situations were presented in a first run lasting 374 TRs, 9 min 21 sec (561 sec), followed by two situations in a second run of 256 TRs, 6 min 24 sec (534 sec). For each situation, participants composed a single paragraph describing the events, thoughts, and feelings and provided situation specific negative self-beliefs (NSBs, see examples in Table 2).

Each situation consisted of an instruction to React (1.5 sec), 16 sentences in white font against a black background describing the situation (3 sec each), 10 NSBs (9 sec each) embedded in the unfolding story in uppercase letters that flashed 9 times (850 msec on + 150 msec off), and a negative emotion rating after each NSB (3 sec) (Figure 1c). Negative self-beliefs were flashed to maintain attention. Participants were trained prior to scanning to react to NSBs by reflecting on how the NSB represents something that is true about themselves.

### Image acquisition

Imaging was performed on a General Electric 3-T Signa magnet with a T2*-weighted gradient echo spiral-in/out pulse sequence [38] and a custom-built quadrature “dome” elliptical bird cage head-coil (GE Healthcare, Milwaukee, Wisconsin). Head movement was minimized using a bite-bar and foam padding. Functional volumes (516 for faces, 684 for criticism, 630 for belief tasks) were obtained from 22 sequential axial slices (repetition time = 1500 milliseconds, echo time = 30 milliseconds, flip angle = 60°, field of view = 22 cm, matrix = 64x64, single-shot, resolution = 3.438 mm^2^ x 4.5 mm). Three-dimensional high-resolution anatomical scans were acquired using a fast spin-echo spoiled gradient recall (resolution = .8594 mm^2^ x 1.5 mm; field of view = 22 cm, frequency encoding = 256).

### fMRI data pre-processing

Analysis of Functional Neuroimages (AFNI) software [39] was used for preprocessing and statistical analysis. Each functional run was subjected to preprocessing steps to maximize signal-to-noise contrast. This included an analysis of potential outliers, volume registration to a base image, motion correction, 4 mm^3^ isotropic Gaussian spatial smoothing, high-pass filtering (.011 Hz), linear detrending, and conversion into BOLD signal percentage change in each voxel. In addition, to allow for T2* equilibration effects, the first four images of each functional run were excluded. For the Criticism and Beliefs tasks, the two functional runs were concatenated prior to statistical analysis. No volumes demonstrated motion in the x, y, or z directions in excess of ±1 mm. There was no evidence of stimulus-correlated motion, as assessed by correlations between condition specific reference functions and x, y, z motion correction parameters.

### fMRI statistical analysis

Multiple-regression implemented with AFNI 3dDeconvolve included baseline parameters to remove mean, linear, and quadratic trends, and motion-related variance in the BOLD signal. For each task, the React condition was defined as: for Faces, the 9 sec single face stimulus; for Criticism, the 7.5 sec. evaluation period; and for the beliefs the 9 sec. of NSBs (see bold lines in Figures 1a,b,c). Regressors for the Asterisks and React conditions were convolved with the Cohen’s gamma variate model of the hemodynamic response function [40]. Functional MRI BOLD signal intensity was computed as percentage of signal change, an effect size measure [(MR signal per voxel per time point / mean MR signal in that voxel for the entire functional run) x 100].

Individual brain maps were converted to Talairach atlas space [41] and second-level group statistical parametric maps were produced according to a random-effects model. To correct for multiple comparisons, AlphaSim, a Monte Carlo simulation bootstrapping program in the AFNI library, was used to protect against false positives [42]. This method uses a voxel-wise and cluster volume joint-probability threshold to establish a cluster-wise false positive cluster detection level. The cluster statistical threshold for the between group analyses consisted of a voxel-wise *P* < .005 and cluster volume higher than 162 mm^3^ (4 voxels x 3.438 mm^3^) to protect against false-positive cluster detection at *P* < .01.

### Regions of interest analyses

**Figure 2:**
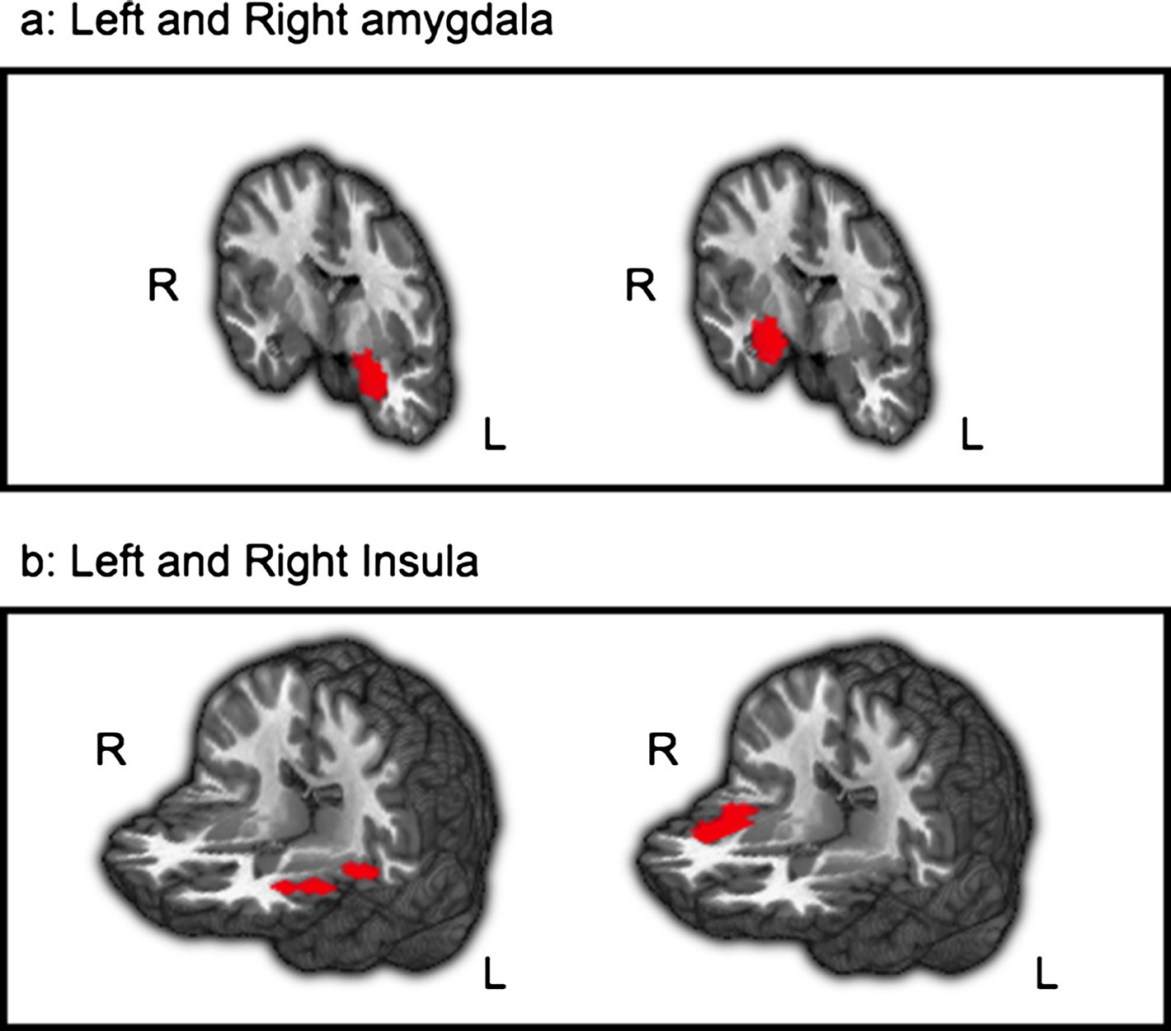
**The a priori regions of interest. a**: Left and right amygdala. **b**: Left and right insula.

To examine emotion reactivity related brain responses, we used the left and right amygdala, and left and right insula as *a priori* regions of interest (ROIs) (Figure 2). The amygdala was anatomically defined using the AFNI Talairach Daemon. The insula ROI was defined as the social-emotional sub-region identified in a meta-analysis by Kurth et al. ([22], see Additional file 1: Table S1 for peak xyz Talairach coordinates). We created spherical masks (radius = 7 mm, volume = 1,437 mm^3^) centered on the peak xyz Talairach coordinates in left and right insula regions, separately. The volume of each ROI was: left amygdala: 81 voxels = 3,292 mm^3^, right amygdala: 81 voxels = 3,292 mm3, left insula: 237 voxels = 9,631 mm3, right insula: 244 voxels = 9,915 mm. We examined BOLD responses within these ROIs with a SAD versus HC between-group ANOVA of React versus Asterisks. The mean and standard deviations per ROI, per group, per task, were calculated. We removed outliers defined as greater than 3 standard deviations from the mean per ROI per group per task.

To examine the relationship between social anxiety symptom severity and brain responses in SAD patients, we computed Pearson correlation coefficients using SPSS. To correct for Type 1 error, the statistical threshold was set at *P* < .02 (*P* = .05/3), to adjust for the three correlations that were tested (symptom severity and brain responses during Faces, symptom severity and brain responses during Criticism, and symptom severity and brain responses during Beliefs).

### Whole-brain analyses

For whole-brain analyses, we identified a sub-group of the 27 SAD patients with the highest social anxiety symptom severity (LSAS-SR scores between 85–102) and compared them to the 27 HC using a between-group ANOVA of React versus Asterisk. We selected the SAD participants with the highest levels of symptom severity to provide the most sensitive test possible of our hypothesis that groups would differ in behavior and brain responses.

## Results

### Primary analyses

#### Faces

##### Negative emotion ratings

**Figure 3:**
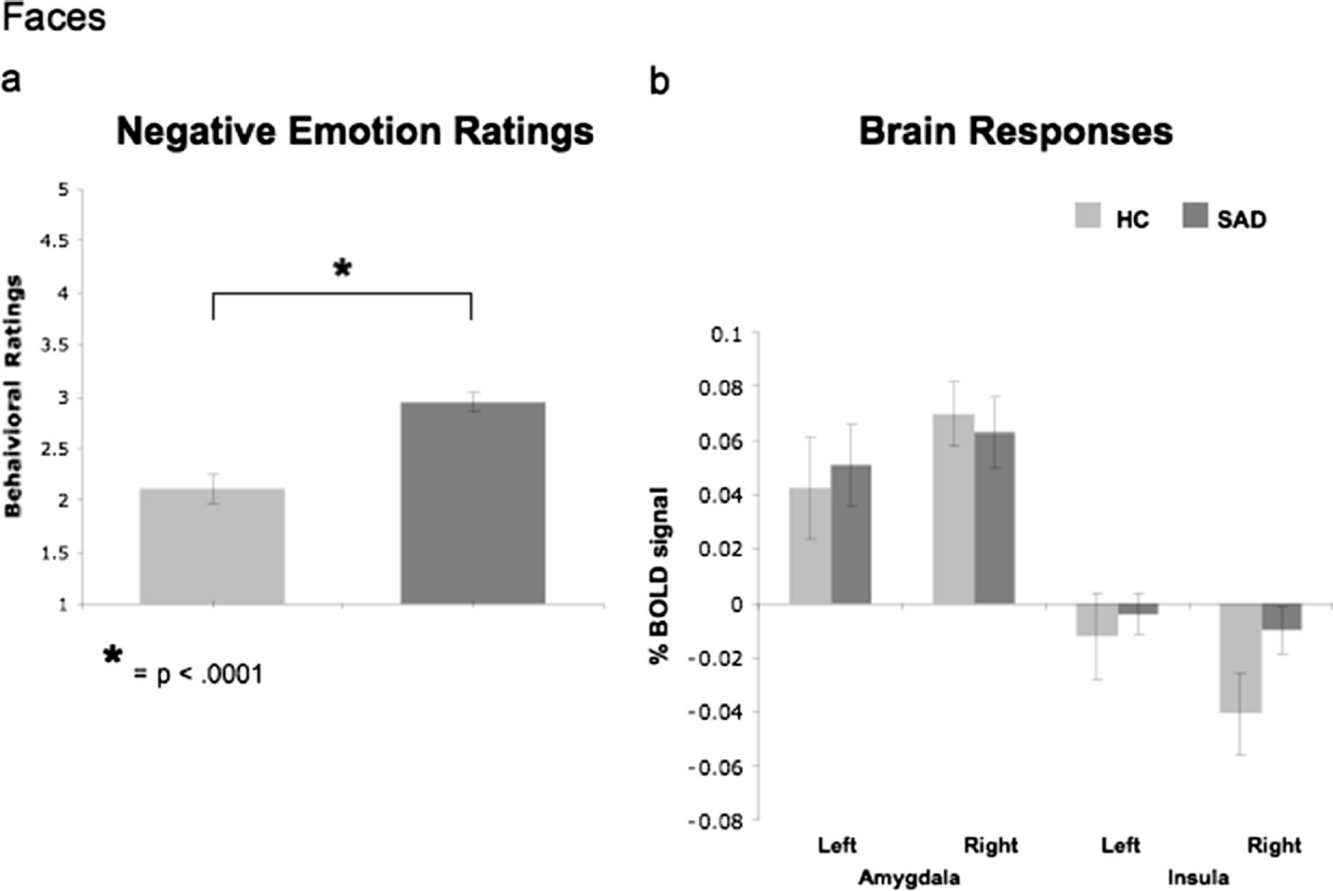
**Faces task. **Between groups differences in negative emotion ratings (**a**) and in % BOLD signal changes in the left and right amygdala and left and right insula (**b**).

A between-group t-test revealed greater negative emotion ratings for SAD (n = 63, mean = 2.94, SD = 0.69) than HC (n = 28, mean = 2.11, SD = 0.82; t_89_ = 4.98, *P* < .0001) when reacting to harsh faces (Figure 3a).

##### Brain responses

For react faces versus asterisks counting, between-group t-tests of BOLD responses showed no differences between SAD and HC in left and right amygdala (all *P*s > .74), and in the left and right insula (all *P*s > .07, Figure 3b). In supplementary analyses, between-group whole brain t-tests showed that, compared to HC, patients had *greater* BOLD responses in the medial frontal gyrus, left middle frontal gyrus, superior frontal gyrus, right superior temporal gyrus, and left middle temporal gyrus/inferior frontal gyrus (Table 3).

**Table 3:** Between-group differential BOLD responses for React versus Asterisks in 27 patients with SAD versus 27 HC

**Brain regions**	**x y z**	**Vol (mm**^**3**^**)**	***t*-value**
**Faces**			
**SAD > HC**			
Medial Frontal Gyrus	0, 62, 8	2235	4.4
Superior Medial Frontal Gyrus	0, 52, 36	447	3.2
Left Medial Frontal Gyrus	−7, 59, -2	244	3.33
	−14, 52, 1	203	3.21
Left Middle Frontal Gyrus	−28, 52, 5	162	3.12
	−28, 49, 12	284	3.11
Left Middle Temporal Gyrus	−52, 14, -12	325	3.6
Right Superior Temporal Gyrus	52, -61, 19	406	3.03
**Criticism**			
**SAD > HC**			
Left Lingual Gyrus	−24, -75, -9	244	3.13
	−17, -82, 5	162	2.96
Left Middle Temporal Gyrus	−52, 0, -16	203	3.45
Right Middle Temporal Gyrus	48, -10, -16	203	3.71
Right Parahippocampus	14, -10, -16	162	3.23
**Beliefs**			
**SAD > HC - none**			
**HC > SAD**			
Right Middle Frontal Gyrus	52, 38, 36	244	3.19
Left Superior Temporal Gyrus	−69, -27, 12	162	3.46

Note. *t*-value threshold ≥ 2.932, voxel *P* < 0.005, minimum cluster volume threshold ≥162 mm^3^ (4 voxels x 3.438 mm^3^), cluster *P* < 0.01.

BA = Brodmann area, xyz = Talairach and Tournoux coordinates of maximum BOLD signal intensity voxel.

#### Criticism

##### Negative emotion ratings

**Figure 4:**
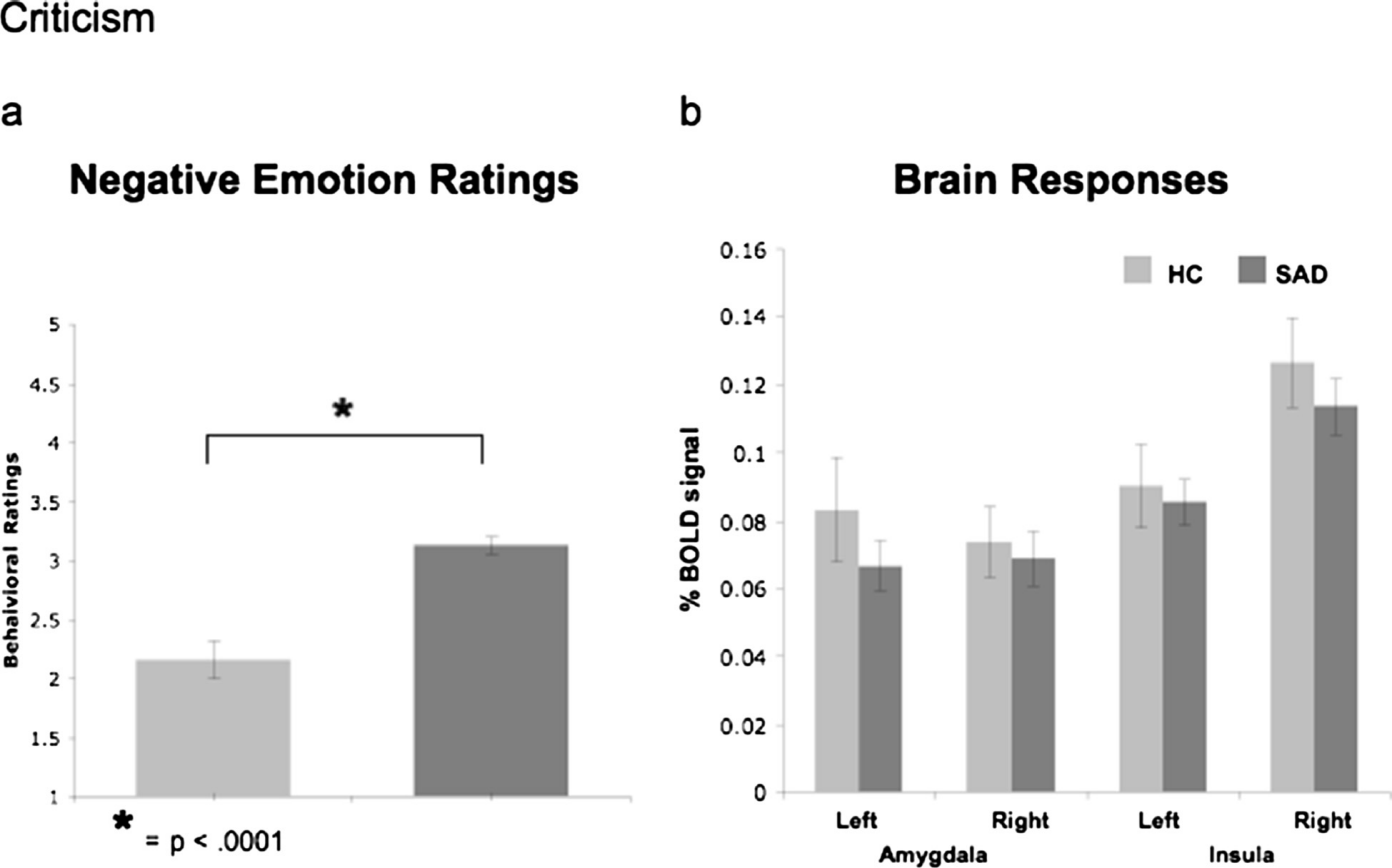
**Criticism task. **Between groups differences in negative emotion ratings (**a**) and in % BOLD signal changes in the left and right amygdala and left and right insula (**b**).

A between-group t-test revealed greater negative emotion ratings for SAD (n = 66, mean = 3.15, SD = 0.81) than HC (n = 28, mean = 2.16, SD = 0.73; t_92_ = 5.57, *P* < .0001) when reacting to criticism (Figure 4a).

##### Brain responses

There were no differences between SAD and HC in left and right amygdala (all *P*s > .28), and left and right insula (all *P*s > .40, Figure 4b). A between-group whole-brain t-test for react to criticism versus asterisks counting showed that, compared to HC, patients had *greater* BOLD responses in the left lingual gyrus, bilateral middle temporal gyrus, and the right parahippocampal gyrus (Table 3).

#### Beliefs

##### Negative emotion ratings

**Figure 5:**
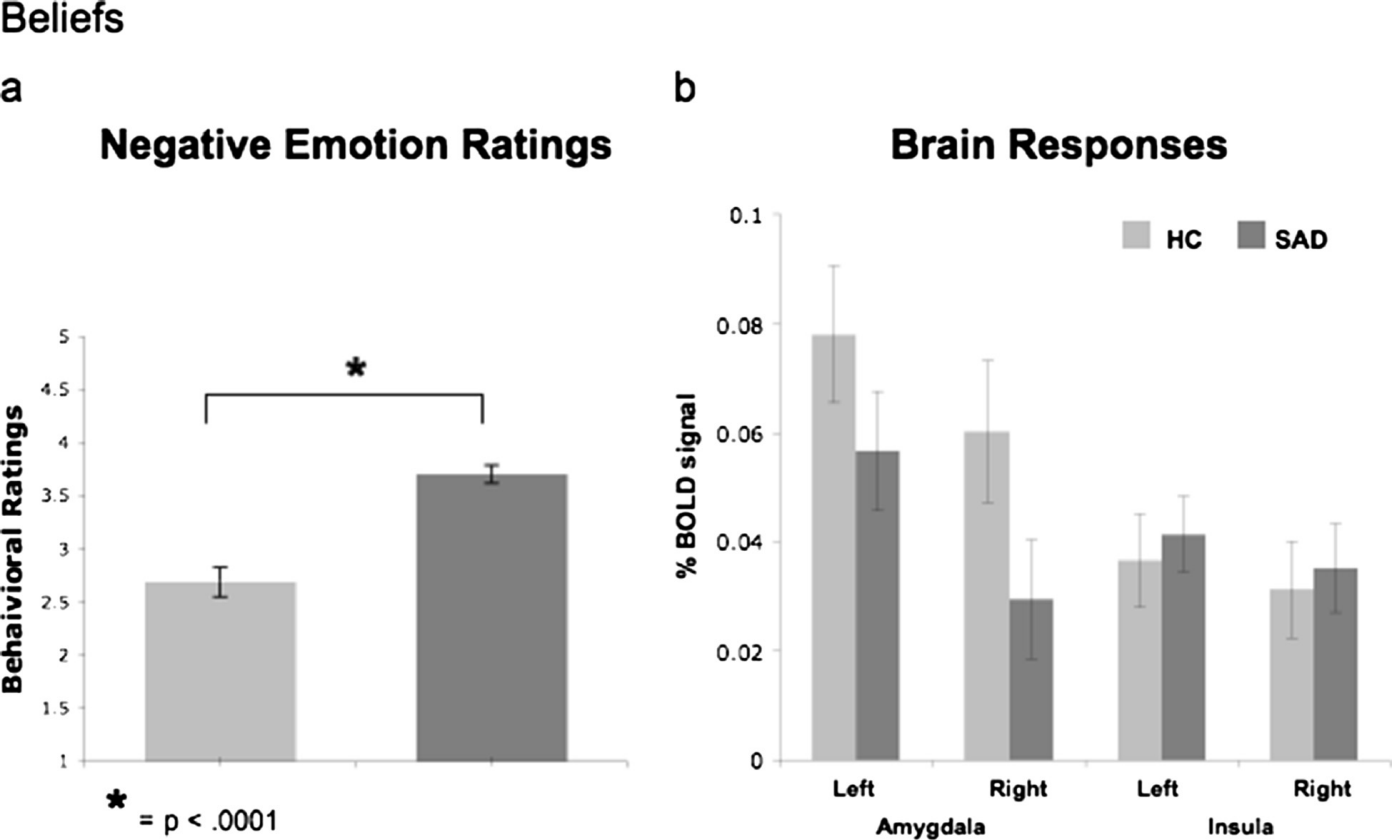
**Beliefs task. **Between groups differences in negative emotion ratings (**a**) and in % BOLD signal changes in the left and right amygdala and left and right insula (**b**).

A between-group t-test revealed greater negative emotion ratings for SAD (n = 65, mean = 3.70, SD = 0.60) than HC (n = 25, mean = 2.69, SD = 1.04; t_88_ = 5.76, *P* < .0001) when reacting to negative self-beliefs (Figure 5a).

##### Brain responses

There were no differences between SAD and HC in left and right amygdala (all *P*s > .12), and left and right insula (all *P*s > .69, Figure 5b). A between-group whole-brain t-test showed that, compared to HC, patients had *lesser* BOLD responses in the right middle frontal gyrus and left superior frontal gyrus when reacting to beliefs versus asterisks (Table 3).

### Secondary analyses

#### Negative emotion ratings

##### Between-group differences

**Figure 6:**
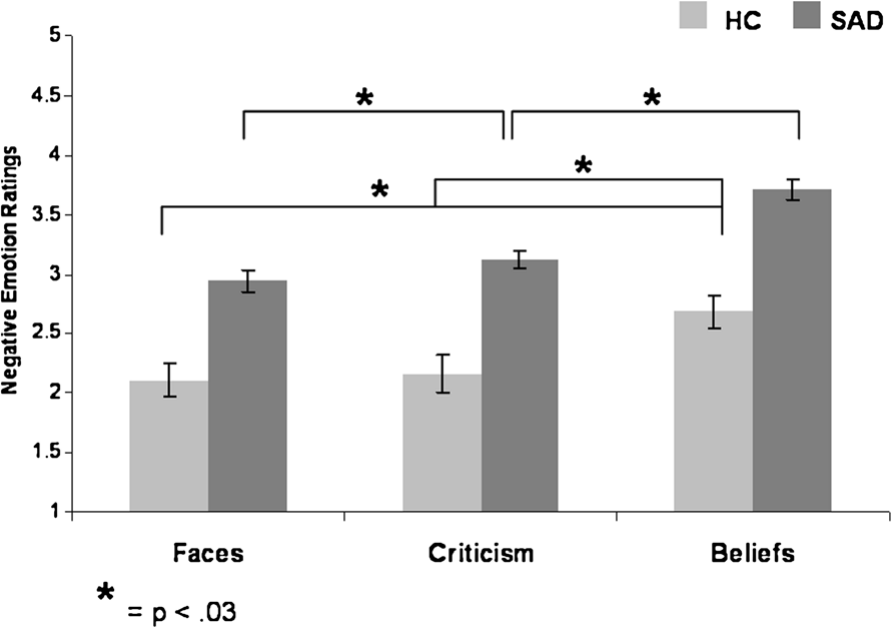
**Task effects within each group - negative emotion ratings. **For SAD, ratings were the highest for Beliefs, and lowest for the Faces task (Beliefs > Criticism > Faces). For HC, ratings were the highest for Beliefs, with no difference between Criticism and Faces (Beliefs > Criticism = Faces).

A 2 Group (SAD, HC) x 3 Task (Faces, Criticism, Beliefs) repeated-measures ANOVA of negative emotion ratings yielded main effects of group (SAD > HC, F_1,85_ = 35.34, *P* < .0001, partial eta^2^ (η_p_^2^) = .29) and task (F_2,170_ = 42.80, *P* < .0001, η_p_^2^ = .34, Belief > Criticism > Faces for SAD; Belief > Criticism = Faces for HC), with no group by task interaction (Figure 6).

##### Association with social anxiety symptoms

In patients with SAD, no correlation was found between social anxiety symptom severity (LSAS-SR) and negative emotion ratings when reacting to faces (*P* > .08), criticism (*P* > .05), and beliefs (*P* > .54).

#### Brain responses

##### Between group differences

To examine the differential responses to each of the tasks in SAD and HC, we conducted a 2 Group (SAD, HC) x 3 Task (Faces, Criticism, Beliefs) repeated-measures ANOVA of BOLD responses in the left and right amygdala and insula.

##### Amygdala

**Figure 7:**
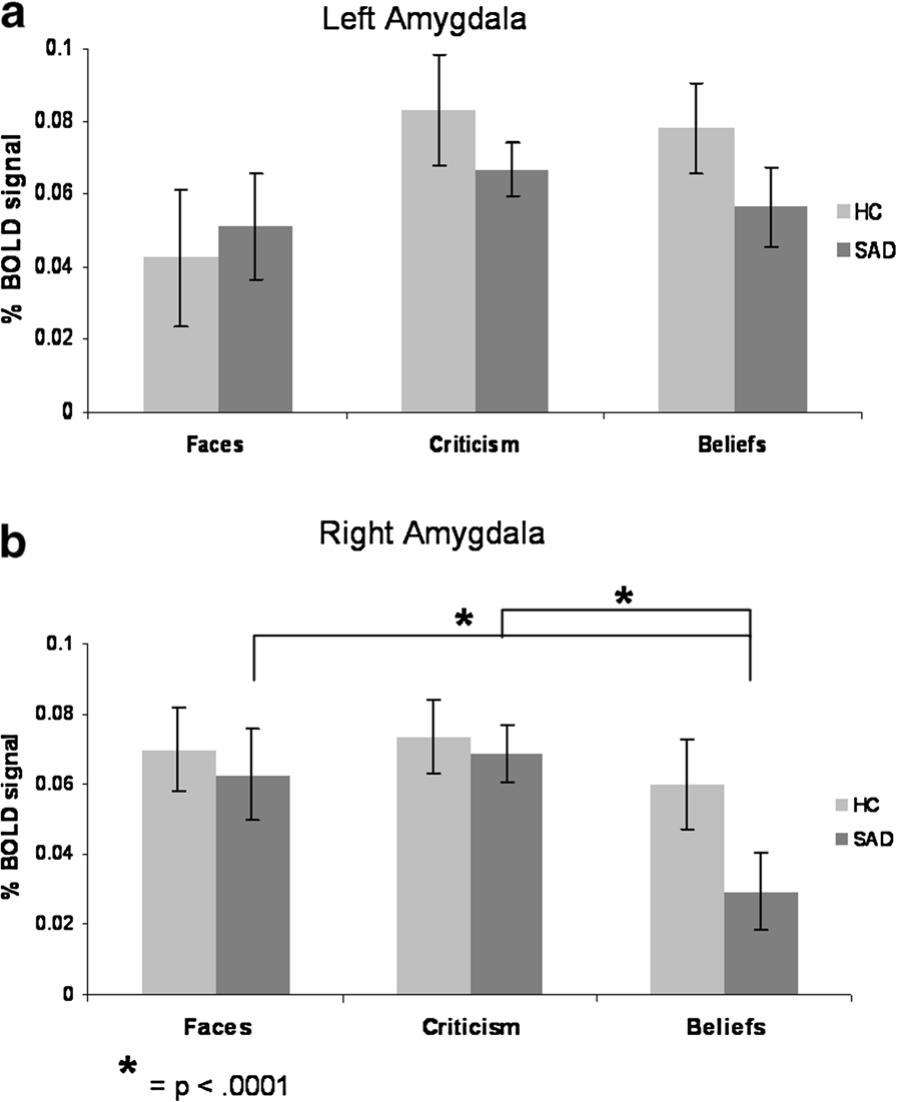
**Task effects within each group – amygdala BOLD Responses. **For the left amygdala, there was no main effect of task in SAD and in HC. For the right amygdala, there were greater BOLD responses when reacting to criticism, and when reacting to faces, compared to beliefs (Criticism = Faces > Beliefs) in SAD, and no significant between tasks differences in HC (Criticism = Faces = Beliefs).

For the left amygdala, there were no main effects of task or group, and no group by task interaction (all *P*s > .05) (Figure 7a). For the right amygdala, there was a main effect of task (*F*_2,154_ = 4.61, *P* < .011, η_p_^2^ = .06), and no main effect of group or group by task interaction (all *P*s > .28). Follow-up paired-samples t-tests indicated that in SAD, there were greater BOLD responses when reacting to criticism, and when reacting to faces, compared to beliefs, with no significant difference between criticism and faces (Criticism = Faces > Beliefs, all *P*s < .0001). In HC, no significant differences were found in the response to criticism, beliefs, and faces (Criticism = Faces = Belief, all *P*s > .21) (Figure 7b).

##### Insula

**Figure 8:**
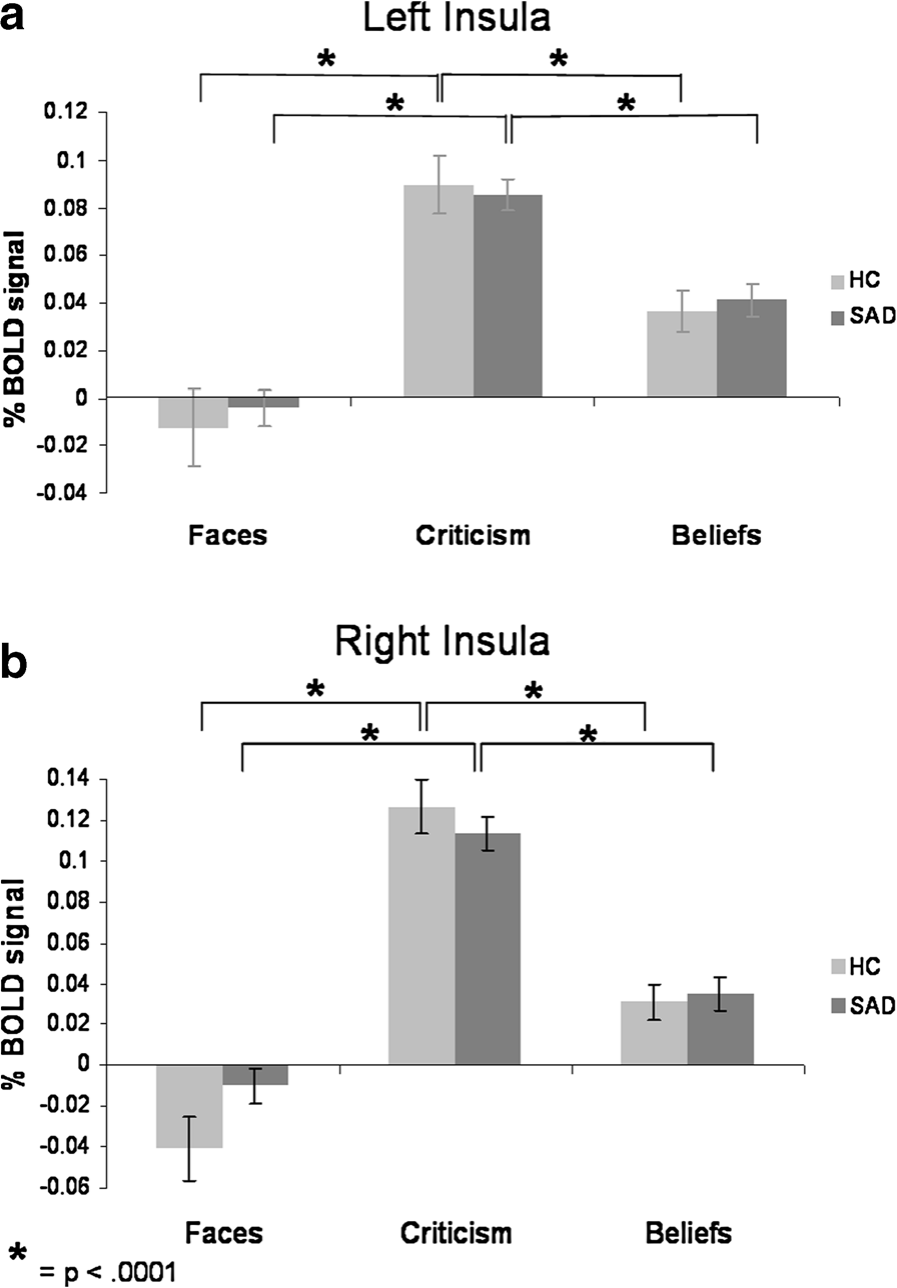
**Task effects within each group – insula BOLD responses. **For both left and right insula, there were greater BOLD responses when reacting to criticism, compared to beliefs, and a significant difference between beliefs and faces, in SAD and HC (Criticism > Beliefs > Faces).

For the left insula, there was a main effect of task (*F*_2,172_ = 76.51, *P* < .0001, η_p_^2^ = .47), and no main effect of group or group by task interaction (all *P*s > .28). Follow-up paired-samples t-tests indicated greater BOLD responses when reacting to criticism, compared to beliefs, and a significant difference between beliefs and faces, in both patients and HC (Criticism > Beliefs > Faces, all *P*s < .0001) (Figure 8a).

For the right insula, there was a main effect of task (*F*_2,174_ = 122.31, *P* < .0001, η_p_^2^ = .58), and a group by task interaction (*F*_2,174_ = 3.97, *P* < .021, η_p_^2^ = .04), with no main effect of group (*P* > .41). Follow-up paired samples t-tests indicated greater BOLD responses when reacting to criticism, compared to beliefs, and a significant difference between beliefs and faces, in both SAD and HC (Criticism > Belief > Faces, all *P*s < .0001) (Figure 8b).

##### Association with social anxiety symptoms

**Figure 9:**
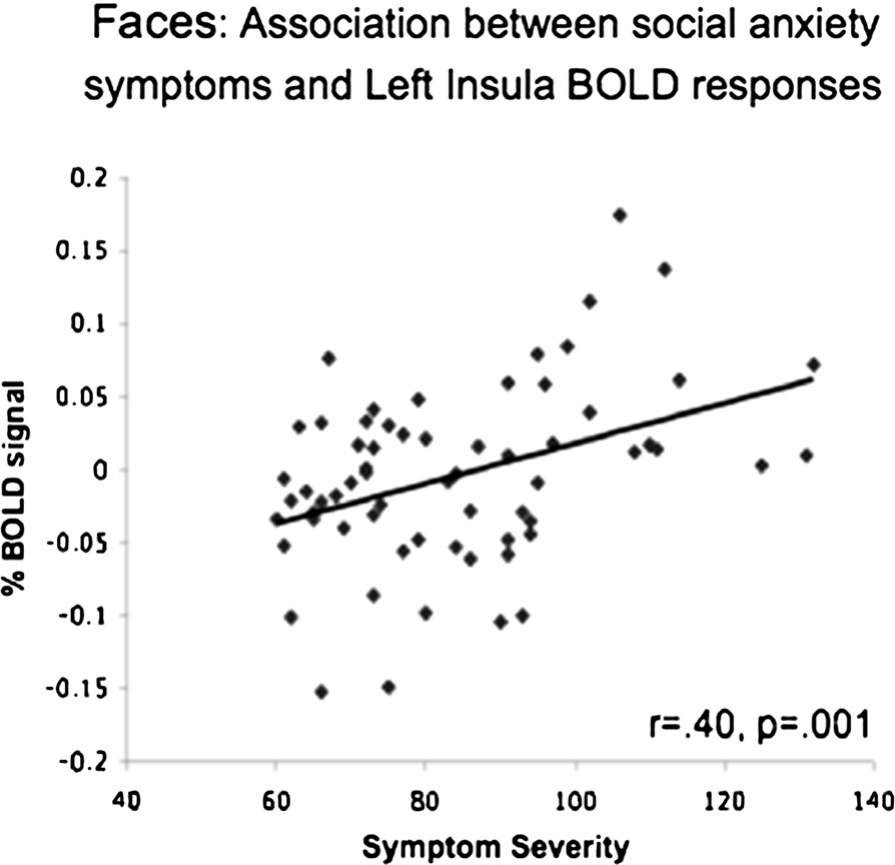
**Faces - association with social anxiety symptoms. **During the Faces task, social anxiety symptom severity was associated with BOLD responses in the left insula.

In patients with SAD, there was an association of social anxiety symptom severity and BOLD responses in the left insula during the Faces task (r = .40, *P* = .001; Figure 9).

## Discussion

The goal of the present study was to investigate behavioral and brain responses in patients with SAD versus HC when reacting to three socio-emotional tasks (faces, criticism, beliefs). As we expected, at the behavioral level all three tasks induced heightened negative reactivity in SAD versus HC participants. However, we found no evidence of between-group differences in amygdala and insula BOLD responses. Both SAD and HC had increased left and right amygdala responses in all three tasks, and increased left and right insula responses in 2 of the 3 tasks (Criticism and Beliefs).

How might this discrepancy between our current results and previous work on emotion-related brain responses in SAD be explained? One possibility is that differences in the experimental tasks used played a crucial role [43]. To date, most studies have used static facial expression stimuli to examine neural processing of interpersonal threat cues. Studies that have investigated social stimuli other than faces have yielded mixed results [16,17,44,45]. For example, no differential recruitment of the amygdala and insula in SAD and HC were found when listening to angry versus neutral voices [17], when performing a social situation task [44], or when playing a ‘decision making’ game with others [45].

In our study, we used three distinct types of socio-emotional tasks with relatively high levels of complexity. The Faces task used a static emotional facial expression with the addition of looming (i.e., the appearance of approaching the perceiver). The Criticism task used dynamic videos of an actor delivering socially critical comments. The Beliefs task used participant-generated negative self-beliefs embedded in an autobiographical script about a socially painful interpersonal situation. In addition, all three tasks paradigms had longer stimulus presentation durations (9–12 sec) than have been used in most prior studies.

It is possible that the increased stimulus complexity and presentation durations in our study increased the likelihood that healthy subjects would evaluate the stimuli as threatening, and thus may have yielded emotion reactivity related brain responses similar to those generated by the patients with SAD. In addition to the stimuli used to elicit emotional responses, other design parameters such as whether the participants are instructed to simply look at the stimuli, or whether they have to perform a task, and whether the task is implicit or explicit, may have contributed to the observed findings [46].

A second possible explanation of our findings builds upon the first, and suggests that at least in the complex socio-emotional contexts we employed in our study (and which characterize many everyday life contexts), increased negative emotion ratings in SAD may be related to exaggerated cognitive biases, including negative self-reflective and ruminative processes. It is also possible that there are differential tendencies to implement spontaneous (uninstructed) emotion regulation directed at the emotion experience in HC versus SAD patients. This could have yielded lower levels of negative emotion ratings in HC. Thus, if the normative pattern shown in HC is to engage in automatic emotion regulation, then one feature of SAD is the absence of such un-cued, automatic activation of emotion regulation. Therefore, maladaptive cognitive processes, such as rumination, suppression, and self-criticism, and ineffective emotion regulation, in SAD may lead to exaggerated negative emotion experience.

If such accounts are correct, we might expect other brain regions (in addition to amygdala and insula) to show differential response patterns between SAD and HC when exposed to socio-emotional stimuli. Indeed, whole brain analyses revealed SAD versus HC differences in several brain regions, specifically, increased frontal, occipital, and temporal cortical activity in SAD versus HC during the Faces and Criticism tasks, and decreased frontal activity in SAD versus HC during the Beliefs task (Table 3). These regions have been shown to be involved in emotion regulation (middle frontal gyrus) [47], recognition of faces (middle temporal gyrus) [48], language processing (superior temporal gyrus), memory (lingual gyrus, parahippocampus), and social cognition (medial frontal gyrus, superior temporal gyrus) [49]. In SAD, though not much discussed, abnormal activity in these regions has been related to difficulties in reasoning [28], mentalizing abilities [45], and impaired perception of self and others [50-52]. Additionally, the whole brain analyses further support our findings of no between-group differences in amygdala and insula activity when reacting to the socio-emotional tasks.

Congruent with these findings, in a recent study, Doehrmann and colleagues (2012) examined the association between responses to cognitive-behavioral therapy for SAD and pre-treatment brain activations to social (facial) and nonsocial (scene) stimuli [53]. They found pre-treatment activity in visual cortical regions correlated with treatment outcome, and no association with treatment outcome in the amygdala, despite its robust activation to all experimental conditions. The authors explained these results by emphasizing dysfunctional emotion regulation, and specifically attentional deployment, in SAD. They suggested that CBT is perhaps particularly successful in patients with better emotion regulation capacities, which is correlated with already stronger responses to angry faces in visual regions. Thus, in addition to showing no abnormality in baseline amygdala response in SAD, this study suggests that brain regions more related to emotion regulation and not to emotion reactivity, might be the core deficit in SAD, and the focus of change during treatment.

More generally, our finding of increased negativity in SAD compared to HC when facing emotional stimuli, with no such SAD versus HC differences in the amygdala and insula responses, could suggest that self-report of negative emotion is less tightly coupled with increased limbic activity than is typically thought, at least in the context of social anxiety. This hypothesis accords with the findings of Mauss and colleagues [32], namely, a decoupling of subjective emotion and physiological measures and objective physiological responses in high and low socially anxious participants when giving an impromptu speech. The authors suggested that the findings of increased physiological activation during the speech in all participants (and not only in participants who reported increased experience of anxiety) leaves open the possibility that physiological activation might be necessary for the experience of anxiety, but such activation is clearly not sufficient to explain inter-individual variation in anxiety experience. The results of the current study strengthen the conclusions of Mauss and her colleagues. It might be that the relation between subjective feeling and physiological response is more complex than a simple ‘amygdala = emotion’ equation would suggest.

It also bears comment that in the current study, examination of between task effects enabled us to examine whether the behavioral and neural results were related to a particular type of socio-emotional stimuli. Although all three tasks were successful in terms of inducing higher emotional responses in SAD, compared to HC, it seems that no task activated all emotion generation brain regions consistently. Our results indicate that different contexts, i.e. different “anxiety-inducing” experimental probes may have quite different effects, behaviorally and neurally. Behaviorally, the Beliefs task yielded the highest negative emotion reactions in both SAD and HC. Thus, the idiographic, participant-generated negative self-beliefs were the most potent emotional probe, most likely because of their self-relevance or personal salience. These probes were also the most ecologically valid type of stimuli in our study, as negative self-focused automatic thoughts are usually the focus of most forms of psychological interventions for SAD. Interestingly, the Beliefs task, which elicited the greatest negative emotion responses, did not elicit the greatest brain responses. Specifically, right amygdala responses were equally high for Criticism and Faces, and lower for Beliefs (with no differential task effects in the left amygdala) in SAD patients. BOLD responses in the left and right insula were highest when reacting to Criticism > Beliefs > Faces in both SAD and HC.

Contrary to our hypothesis, social anxiety symptom severity was not correlated with negative emotion ratings, and was associated only with greater left insula activation during the Faces task, with no association with amygdala response. This finding accords with the findings of Schmidt and colleagues [46] of a positive correlation between insula activity and symptom severity in SAD patients, despite no differential effect in the insula between SAD and HC, during explicit processing of verbal threat-related stimuli. The association with symptom severity suggests that within patients with SAD, symptom severity matters. Thus, during the Faces task, even though as a group SAD patients had insula responses comparable to the HC group, greater social anxiety symptom severity within patients was found to be associated with increased neural responses. A recent study by Furmark and colleagues further supports the importance of looking beyond only meeting diagnostic criteria for SAD. They found that both patients with SAD and HC had increased left amygdala activation in response to angry compared with neutral faces, but that genotype (serotonergic polymorphisms) and not diagnosis explained a significant portion of the variance in amygdala responsiveness [54].

## Conclusions

This is the first study to specifically assess three distinct types of socio-emotional experimental tasks in a large sample of patients with SAD at a single time point. Our main finding is similar increased amygdala and insula responses when reacting to socio-emotional stimuli, despite increased emotional responses in SAD, compared to HC. The fact that we obtained the same result using three different tasks provides strong validation of our findings. To test our hypothesis of deficits in emotion regulation processes in SAD, future studies should investigate regulation processes, both explicit (when instructed to regulate) and implicit (spontaneous), in SAD and in HC. A direct examination of emotion regulation processes and their association with neural activity in limbic and cortical brain regions could help in understanding the deficient appraisal processes in SAD.

Methodologically, more studies are needed to further examine different kinds of tasks/contexts, which might help to evaluate the influence of different variables, such as the degree of personal salience, the intensity of the emotional stimuli, and the modality of the stimuli, on the behavioral and neural responses. Also, in addition to examination of negative emotional stimuli, an interesting venue could be the investigation of positive emotional stimuli, such as social praise, or positive self-related thoughts.

In this study, in order to be able to compare between the different tasks, we contrasted the React condition to an Asterisks condition. However, in the future, it will be important to examine the results when contrasting reactivity to negative stimuli with reactivity to neutral stimuli as well.

Finally, an interesting question arising from the current results is whether other clinical populations such as major depression, generalized anxiety, or mixed anxiety-depression, show the same dissociation between heightened emotional reactivity and similar limbic responses, compared to HC. In the future, it will be important to compare behavioral and BOLD responses during negative emotional contexts in SAD group versus other clinical populations, in order to examine the specificity of the results reported here.

## Competing interests

The authors of this manuscript do not have any direct or indirect conflicts of interest to disclose.

## Authors’ contributions

MZ participated in the study’s design and coordination, contributed to data acquisition, conducted the fMRI sessions, conducted the data analyses, and took lead on writing the manuscript. PRG helped conceive of the design of the study, participated in the study’s design and coordination, contributed to data acquisition, conducted the fMRI sessions, consulted on data analyses, and contributed to writing the manuscript. HJ participated in the study’s design and coordination, contributed to data acquisition, conducted the fMRI sessions, consulted on data analyses, and contributed to writing the manuscript. KSH consulted on data analyses, and contributed to writing the manuscript. JJG helped conceive of the design of the study, consulted on data analyses, and contributed to writing the manuscript. All authors have read and approved the final manuscript.

## Supplementary Material

Additional file 1: Table S1 Peak XYZ Talairach Coordinates for the Left and Right Insula, as Defined by Kurth’s Meta Analysis [22]. Click here for file
