# Supplementary material for: Is there less to social anxiety than meets the eye? Behavioral and neural responses to three socio-emotional tasks
Source: Biol Mood Anxiety Disord. 2013 Mar 1;3:5. doi: 10.1186/2045-5380-3-5 (PMC3608942; doi:10.1186/2045-5380-3-5)
Supplement: Additional file 1: Table S1 — Peak XYZ Talairach Coordinates for the Left and Right Insula, as Defined by Kurth’s Meta Analysis [22]. [file 2045-5380-3-5-S1.doc]

Supplementary Table 1: Peak XYZ Talairach Coordinates for the Left and Right Insula, as Defined by Kurth’s Meta Analysis [22].

| **Left Insula** | x y z |
| --- | --- |
| **Emotion** | −31 24 -4 |
|  | −30 12 10 |
| **Empathy** | −40 15 3 |
|  | −31 16 -19 |
|  | −41 -7 4 |
| **Right Insula** | x y z |
| **Emotion** | 42 15 -3 |
|  | 39 7 0 |
|  | 28 17 -15 |
| **Empathy** | 39 19 3 |
|  | 37 8 7 |
|  | 46 -6 -1 |
